# Molecular Property Prediction of Modified Gedunin Using Machine Learning

**DOI:** 10.3390/molecules28031125

**Published:** 2023-01-23

**Authors:** Mohammed Aly, Abdullah Shawan Alotaibi

**Affiliations:** 1 Department of Artificial Intelligence, Faculty of Artificial Intelligence, Egyptian Russian University, Badr City 11829, Egypt; 2 Computer Science Department, Shaqra University, Shaqra City 11961, Saudi Arabia

**Keywords:** CNN, LSTM, RNN

## Abstract

Images of molecules are often utilized in education and synthetic exploration to predict molecular characteristics. Deep learning (DL) has also had an influence on drug research, such as the interpretation of cellular images as well as the development of innovative methods for the synthesis of organic molecules. Although research in these areas has been significant, a comprehensive review of DL applications in drug development would be beyond the scope of a single Account. In this study, we will concentrate on a single major area where DL has influenced molecular design: the prediction of molecular properties of modified gedunin using machine learning (ML). AI and ML technologies are critical in drug research and development. In these other words, deep learning (DL) algorithms and artificial neural networks (ANN) have changed the field. In short, advances in AI and ML present a good potential for rational drug design and exploration, which will ultimately benefit humanity. In this paper, long short-term memory (LSTM) was used to convert a modified gedunin SMILE into a molecular image. The 2D molecular representations and their immediately visible highlights should then provide adequate data to predict the subordinate characteristics of atom design. We aim to find the properties of modified gedunin using K-means clustering; RNN-like ML tools. To support this postulation, neural network (NN) clustering based on the AI picture is used and evaluated in this study. The novel chemical developed via profound learning has long been predicted on characteristics. As a result, LSTM with RNNs allow us to predict the properties of molecules of modified gedunin. The total accuracy of the suggested model is 98.68%. The accuracy of the molecular property prediction of modified gedunin research is promising enough to evaluate extrapolation and generalization. The model suggested in this research requires just seconds or minutes to calculate, making it faster as well as more effective than existing techniques. In short, ML can be a useful tool for predicting the properties of modified gedunin molecules.

## 1. Introduction

The prediction of molecular characteristics in the realm of drug development is an important effort. Computer approaches, due to their accurate forecasting, can speed up the whole interaction between finding better applicants in a faster, cheaper manner. This is particularly convincing when you consider that the usual cost of improving a drug is now estimated to be approximately USD2.8 billion [1]. The usual technique of using silicon for predicting molecular characteristics depends mostly on the removal of fingerprints or hand-made highlights, which are subsequently used in combination with AI computation. In conclusion, this kind of atomic portrayal is unilaterally presented by space professionals, to gather the highlights essential for the task to be carried out [2]. To advance beyond that type of tendency to a broader approach, several forms of IAs were incorporated into the field of prediction of molecular properties. The calculation of deep learning has resurged in particular, due not only to the acceleration of computer power and the increase in accessibility of large information indices but also to the vast scale of its performance, for instance, in the field of characteristic language use [3] and example recognition [4]. These networks are mechanized to study representations for a certain task and can thus eliminate the confused design interaction of the components [5]. A suitable portrait for molecules should be created to use deep learning calculations and cover the explicit component design region. As molecules may be seen as diagrams, one technique just involves using an atomic graph portrait—resulting in the improvement of neural graphic networks (GNNs) which have become increasingly commonplace in recent years [6,7,8]. The achievements in the development of traditional AI techniques, in particular, with expectations of quantum mechanical properties [5,9,10,11,12], physicochemical properties, as well as hydrophobicity, [13,14,15] and predictions of toxicity, appear to give perhaps the most encouraging profound learning strategies in explicit diagram tasks. Due to the rapid speed growth of the distributions recognized with GNN expectations for molecular properties, it may sometimes be difficult to know the current situation in this sector.

A deep study into synthetic scanning becomes more important at a speedy rate and when the prediction of particle characteristics, such as free solvent energy [16] or ionizing energy [17], contends with or even outstrips existing abdominal muscle calculations. The range of potential AI (ML) calculation errant appears endless and can range from a grouping of bioactive mixtures into dynamic and non-dynamic HIV replication classifications [18] to retrograde assignments in which a particular value, such as lipophilicity, is predicted, depending on the information [19]. However, ML computations are not limited to class and quality forecasts. They can generate new compound buildings depending on input structures or desired communication goals. They can thus maintain the plan of new molecules in the fields of materials and restorative chemistry [20,21,22]. Clustering further evaluates the partitioning of huge nuclear datasets into the most highly harmonic collections and can be utilized from therapeutic research to identify innovative lead structures to speeding up the selection of your PC [23]. Clustering of K-Means is an unmonitored learning-technique grouping the unmarked data into several clusters. Here, K determines the number of predefined clusters that have to be generated, if K = 2, two clusters would be created, and for K = 3, three clusters would be created, and so on.

The usage of strings is a simple way of addressing a subatomic design. A string is a number and letter progression that is used routinely to enhance the determination of subatomic information line transit (SMILES). In these strings, the SMILES string ‘CCCO’ would be used to depict each particle with its edge iotas and neighboring bonds, and an atom such as n Propanol [24]. As the chirality was not properly represented, the IUPAC offered an alternative string depiction called the International Chemical Identifier (InChI) which could not be easily interpreted by a chemist, apart from the focused chiral and the tautomerism [25].

Sub-atomic descriptors are another method to address a sub-atomic design. These descriptors may be direct, directly available from the construction, with highlights such as the number of iotas, the number of heteroatoms, or the subatomic load to establish or reconstruct real characteristics such as the second dipole and the incomplete loading dramatically [26]. These characteristics are then combined into a fixed-length vector in a described request. Additional features such as subatomic fingerprints are available and are typically used. An atomic fingerprint is a longitudinal vector that deals with each component.

In the study of Tang et al. [27], the suggested model was used to predict AASC compressive strength. The ANN model took into account the complicated and nonlinear relationship between compressive strength and concrete components, such as raw materials used in the preparation of the concrete specimen. The relation between both variables and the AASC compressive strength was inverse, with strength increasing but decreasing with a rise in SS. The provided model was further justified as exhibiting high efficiency in predicting strength properties by comparing it to current models.

In the study of Yakub et al. [28], the suggested model was used to discuss the impacts of crystallite size on the performance of copper and iron oxides in reducing NOx, as well as the construction of a prediction model that links crystallite size with H2-SCR efficiency. Monometallic as well as bimetallic catalysts doped over palm kernel shell-activated carbon were explored. Aside from chemical analysis, two predictive equations for NOx conversion and N2 selectivity were established using the ANN approach. The presented model was most relevant for working temperatures ranging from 250 to 300 °C, whereas lower temperature ranges would need additional data.

In the study of Abhyankar et al. [29], the suggested model employed Envisat ASAR VV polarized data and an ANN to detect flooded regions caused by a cyclonic storm. This study solely covered nine mandals in the Guntur district of Andhra Pradesh to identify the flooded regions. The outcomes revealed a high degree of classification accuracy and implied that this might be a quick tool for damage estimates and post-disaster relief and recovery activities.

In the study of Etu et al. [30], the accuracy of the Radial Basis Function Neural Network (RBFNN) as well as the Multiple Linear Regression model (MLR) in forecasting home-based visits was evaluated. The datasets for the study were obtained from a household travel survey in Akure, Nigeria, which used the SPSS 22.0 statistical tool. This study proved the better accuracy of the RBFNN in creating trip-generation forecasts in the study region, and as a result, it was suggested for scientists in carrying out such forecasts.

## 2. Results

**Figure 1:**
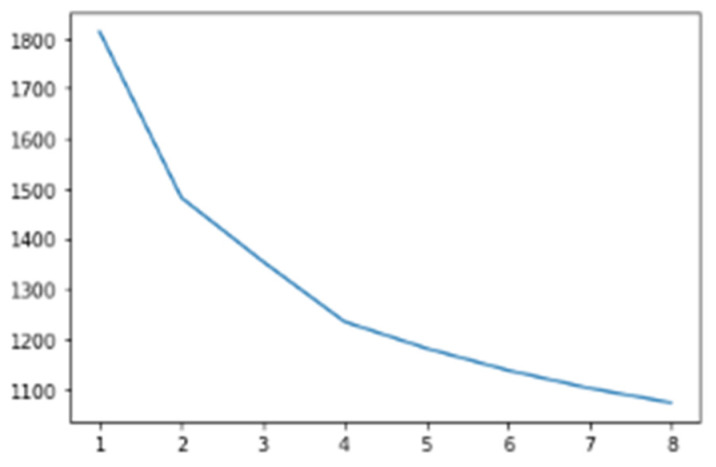
Curve Elbow to determine number of clusters.

**Figure 2:**
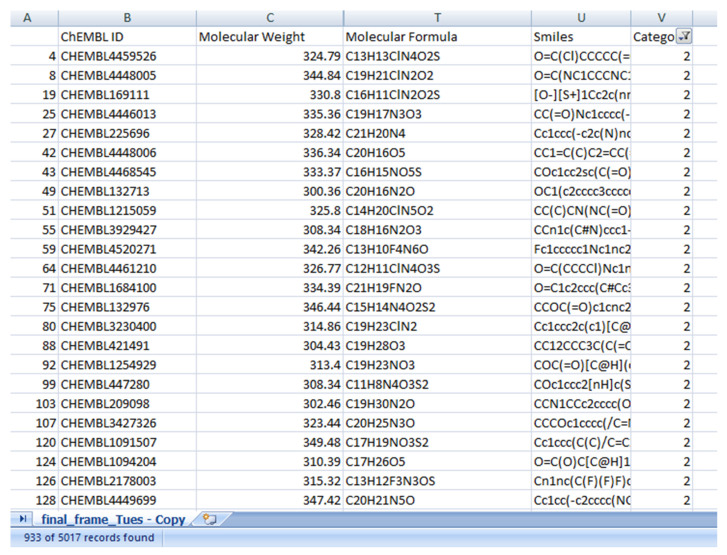
Similar property compounds in cluster 2.

**Figure 3:**
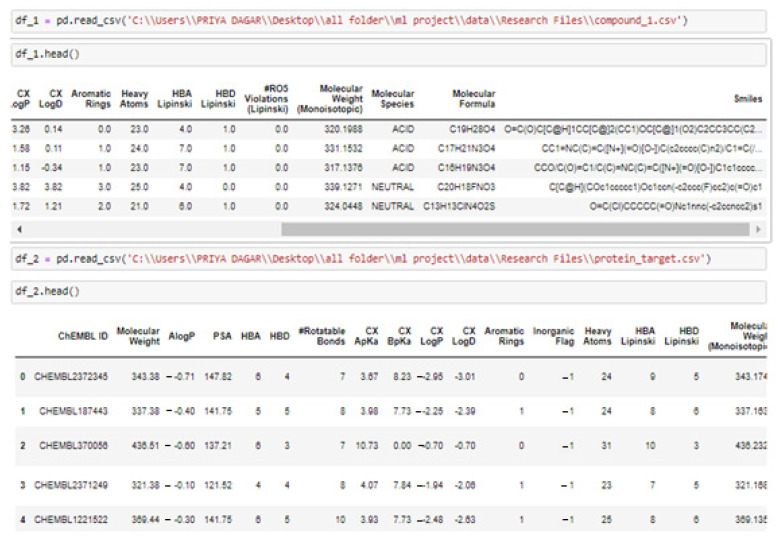
Dataset for training and validation.

LSTM was used to convert the modified gedunin SMILE into an image, which was further classified in cluster 2. In this paper, Curve Elbow was applied to determine the number of clusters (Figure 1). Similar property compounds in cluster 2 included chembl id, molecular weight, molecular formula, and SMILE (Figure 2). This classification is executed by K-means clustering. Based on a silhouette score, four clusters were determined to split molecules into clusters based on common functions or properties using features of a large dataset (Figure 3) as a training and validation set. Modified gedunin shares properties with 933 compounds as shown in (Figure 2). Properties are interleukin-23 receptor inhibitor, adenosine kinase and mitotic spindle assembly protein MAD2B inhibitor, antiproliferative activity, cytotoxicity against human HCC366 cells, anticancer activity against human T47D cells, and growth inhibition of human MCF7 cells, antiprogestational activity, anti-inflammatory activity and anti-tumor activity.

**Figure 4:**
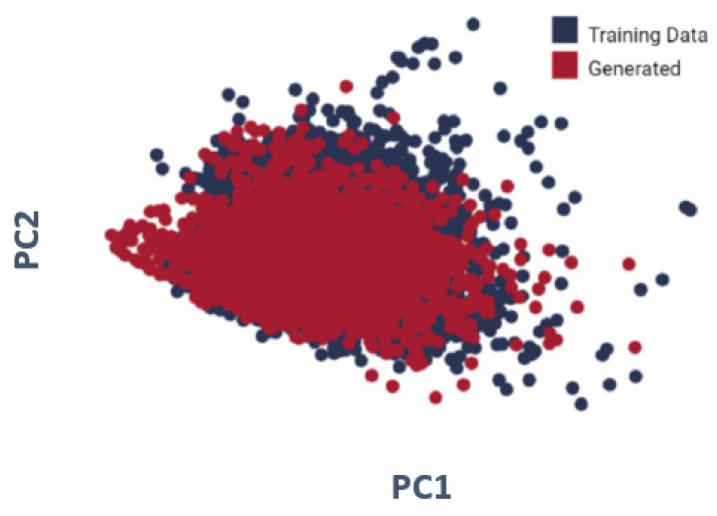
The PCA projection of molecular descriptors from training data as well as molecules created by the LSTM.

We used principal component analysis (PCA) to illustrate the ten properties (interleukin-23 receptor inhibitor, adenosine kinase, and mitotic spindle assembly protein MAD2B inhibitor, antiproliferative activity, cytotoxicity against human HCC366 cells, anticancer activity against human T47D cells, growth inhibition of human MCF7 cells, antiprogestational activity, anti-inflammatory activity, and anti-tumor activity). As shown in Figure 4, for every one of the molecules created by the LSTM as well as randomized molecules from the training set, four molecular descriptors have been examined. To display the data, PCA was performed for dimension reduction, with produced molecules in red as well as training data molecules in blue. The distributions are nearly identical.

The LSTM was utilized to produce the SMILE of modified gedunin. Furthermore, the LSTM focuses on the most optimum region of the chemical space. We also separately displayed the distributions of the ten attributes. Molecules formed in the last transfer-learning iteration have minimized the objective values to levels significantly lower than molecules generated before transfer learning; hence, the model not only concentrates on, but also identifies new, optimum parts of the chemical space.

Several ML models that use various algorithms detect images with high accuracy but become ineffective when processing large quantities of data. To classify the target cell, initially train the ML model so that it can recognize the cell as well as its features, which is achieved by contrasting the image of the targeted cells, which isolates it from the background. Having sufficient data is critical to the accuracy of prediction models. Given a variety of parameters, neural models need a large quantity of training data in order to acquire an appropriate chemical representation and, eventually, the prediction objective itself. Accuracy is used in classification problems to assess the percentage of correctly predictions made by a model. In ML, the accuracy score is an assessment metric that relates the number of correct predictions by a model to the overall number of predictions made. Equation (1) is used to measure the accuracy by dividing the number of correct predictions by the total number of predictions [31,32]:

(1) $$\mathrm{Accuracy}=\frac{Number\;of\;correct\;predictions}{Total\;number\;of\;predictions}\;$$

The total accuracy of the suggested model was 98.68%. The accuracy of the molecular property prediction of modified gedunin research is promising enough to evaluate extrapolation and generalization.

## 3. Materials and Methods

### 3.1. Data Collection and Preprocessing

**Figure 5:**
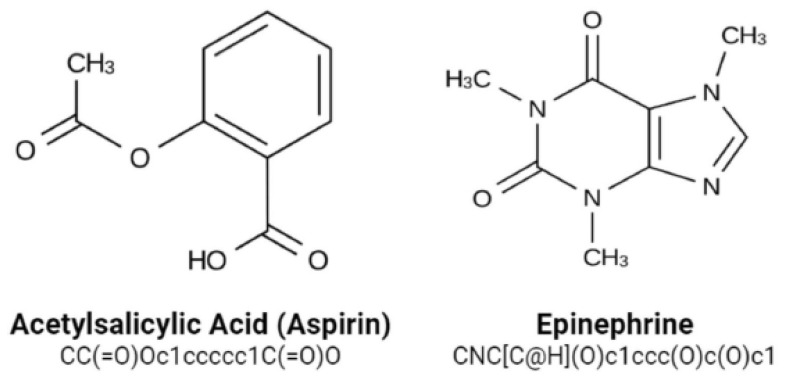
SMILES notations for numerous molecules.

With the CHEMBL and Drug Bank datasets, all relapse businesses were conducted and the Python code under the Rkdit environment was used to transform the SMILES texts to graphic pictures as part of the data pre-processing. ChEMBL is an open data resource that contains information on binding, functional, and ADMET properties for a large number of drug-like bioactive chemicals. On a routine basis, these data are manually extracted from the main published literature, then selected and standardized to enhance their quality and usability across a wide range of chemical biology as well as drug-discovery research concerns. The CHEMBL database now includes 5.4 million bio-activity measurements for over 1 million chemicals as well as 5200 protein targets. The web-based interface, data downloads, and online services are accessible at: https://www.ebi.ac.uk/chembldb (accessed 20 March 2022). The SMILES string format was used to identify molecules for simple interpretation by the RNN model we use. SMILES was built with grammatical consistency as well as machine friendliness in mind, with characters representing atoms, bonds, and chemical structures (Figure 5) [33]. These molecules ranged in length from 35 to 75 characters. These SMILES molecules were encoded in a single pass so that each SMILES character has been defined by a 53-dimensional vector of zeros with a one in the relevant index of the character. These data were then applied to train an RNN, which was then applied to construct acceptable molecules.

### 3.2. Feature Extraction and Clustering

VGGNet is a revolutionary neural network design, that was exhausting features and was proposed by Andrew Zisserman and Karen Simonyan at Oxford University in 2014. In order to cluster data in needed groupings based on a similarity index, the K-mean clustering method was employed. Using the Elbow technique, the cluster number was determined.

We only used this model as a feature extractor, which means we removed the last (prediction) layer to generate a feature vector. We specified the target size to (53, 53) when loading the molecular images since the VGG model requires the molecular images it receives to be 53 × 53 NumPy arrays. We were then able to manually load the VGG model as well as delete the output layer. This indicated that the new final layer was completely connected. Now that the last layer was removed, we used the prediction technique to obtain our feature vector. We utilized this feature_extraction function to extract features from all of the molecular images and saved them in a dictionary. Then, we reduced the dimensions of the feature vector to a much smaller number. We clustered our molecular images next because we had a reduced feature set. The K-mean clustering approach would then enable us to classify our feature vectors into k clusters (k = 4). Therefore, every cluster must contain related molecular images. This allowed us to classify the molecular images into clusters. We were able to see that our model did a good job of clustering the molecular images.

### 3.3. Molecular Property Prediction of Modified Gedunin

**Figure 6:**
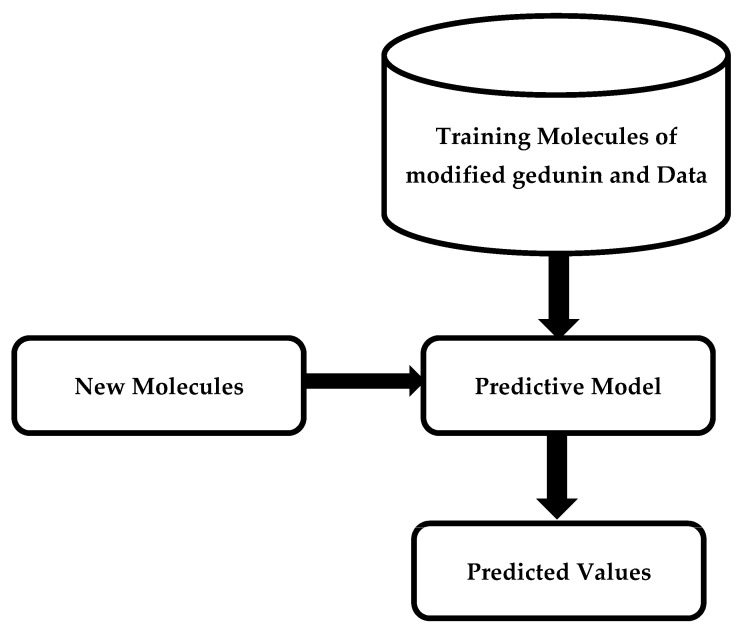
A diagram illustrating the predictive modelling method.

The properties of modified gedunin were anticipated based on the similarity index by predicting the cluster it fits. The idea of virtual screening has fueled decades of study into ML approaches for predicting molecular characteristics varying from bioactivity to bio-distribution and physical features. If effective, these approaches have the potential to change the discovery process by decreasing the time and cost involved with experimental screening while expanding the chemical space that may be investigated. Although traditional testing screening methods may potentially examine millions of molecules, virtual screening may evaluate billions in a short amount of time. Comparable to how medicinal chemists evaluate molecules, the techniques employed in virtual screening try to understand the link between distinct chemical substructures and the target features. The idea is that when given huge quantities of training data in the form of molecules with detected properties, these techniques will be able to systematically catch complex features that people may miss. Figure 6 illustrates an outline of machine learning techniques for predicting molecular properties. A training dataset of molecules and accompanying data is applied to design a model that predicts a collection of test observables.

### 3.4. Neural Networks for Property Prediction

Advances in neural models have created the opportunity of changing this current state of the art. One of the most significant differences between these models is how molecules are handled within the ML technique. The accuracy of the extracted features, which might not be ideal for a specific predictive modeling purpose, is critical to the performance of these models. Neural models, on the other hand, learn their expert feature representations directly from data, converting a molecular graph into a dense continuous vector. Graph convolution, a method borrowed from earlier research in image processing, is indeed a frequent strategy for such translation. While this vector’s coordinates do not often capture human attributes, they have already been proven to be quite adaptable and capable of capturing complicated relations given enough data. The Tox21 Challenge, in which groups employed a range of ML algorithms to predict the outputs of experimental toxicity assays, was an early demonstration of DL effectiveness in drug development.

### 3.5. Data Requirements for Accurate Property Prediction

Access to sufficient data is critical to the accuracy of prediction models. Assuming a wide range of parameters, neural models need a large quantity of training data in order to acquire an appropriate chemical description and, eventually, the prediction problem itself. Unfortunately, obtaining a high sample size may not be possible in many drug-development settings. A regular pharma-lead optimization attempt, which produces only a few thousand molecules does not always create enough data to train a neural model. Furthermore, even a huge training set does not really ensure that the model will effectively adapt to a new drug space. The greater the difference from the initial training set, the more difficult it is for the model to detect the goal attribute properly. The available approaches for estimating confidence cannot accurately estimate the possibility of the model prediction being right.

### 3.6. Uncertainty Quantitation

When an ML model is applied to identify a molecule’s biological activity or physical attributes, it is essential to recognize the prediction’s uncertainty. Although it is reasonable to assume that predictions on molecules identical to those in the training set might be more definite, the field is still unable to agree on effective techniques for quantifying uncertainty. Even as the field progresses toward more complicated learned representations, such as those outlined in Section 3.4., quantifying molecular similarities as well as assessing uncertainty in ML models becomes more complex. Hirschfeld et al. [34] evaluated multiple strategies for uncertainty quantification in NN models using 5 benchmark datasets. The ability of numerous established methodologies to quantify the inaccuracy in model predictions was investigated by these authors. The authors reported that none of the strategies studied could estimate uncertainty consistently across datasets.

### 3.7. Recurring Neural Network (RNN)

RNNs have demonstrated success in modeling sequential data, which is typically encountered in natural language processing (NLP). RNNs may recognize the meaning of data in addition to capturing the data’s grammatical structure. RNNs can always be thought of as several clones of the exact same NN, each one sending input to its successor via a hidden state. By factoring in this hidden state, each NN provides probabilities to the next item in the sequence given all those that came before it. Assuming network parameters ρ, the probability that the whole sequence $S=s_{1}\;s_{2}\;s_{3}\ldots \;s_{N}$ of size N time steps is as follows:

$$P_{\rho }\left(S\right)=P_{\rho }\left(s_{1}\right)P_{\rho }\left(s_{2}|s_{1}\right)P_{\rho }\;\left(s_{3}|s_{1}s_{2}\right)\ldots P_{\rho }\;(s_{N}\;|s_{1}\ldots s_{N-1})$$

Recent neural networks use training data to learn, such as feedforward and convolutionary neural networks (CNNs). They are characterized by their “memory” since they use previous inputs for information to impact the present input and output whereas the artificial recurrent neural network (RNN) architecture utilized in deeper learning is the long short memory (LSTM). In contrast to the typical neural networks for feedforward, LSTM contains feedback connections. Not only single data points (such as pictures) but also whole data sequences may be processed (such as speech or video). LSTM is appropriate for applications such as unsegmented, connected handwriting, voice recognition, and network traffic anomaly or IDS detection (intrusion detection systems), and the visual geometry group VGGNet architecture is ILSVR2014’s first classifying architecture whereas GoogLeNet is the winner. This explains why VGGNet is built up on the top of this architecture by many contemporary model classifications.

### 3.8. Long Short-Term Memory (LSTM)

**Figure 7:**
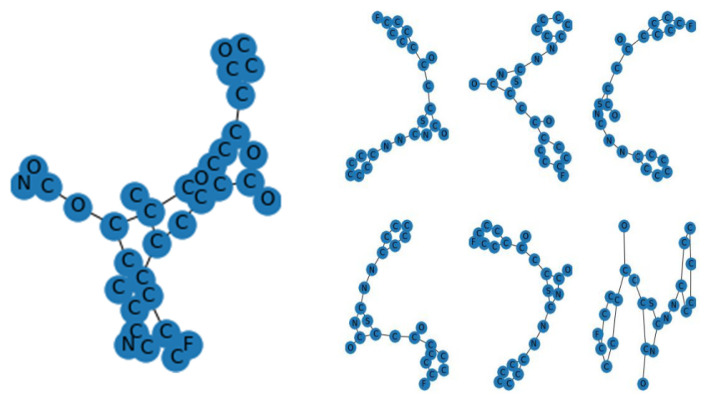
Molecular image of newly generated SMILES using LSTM.

**Figure 8:**
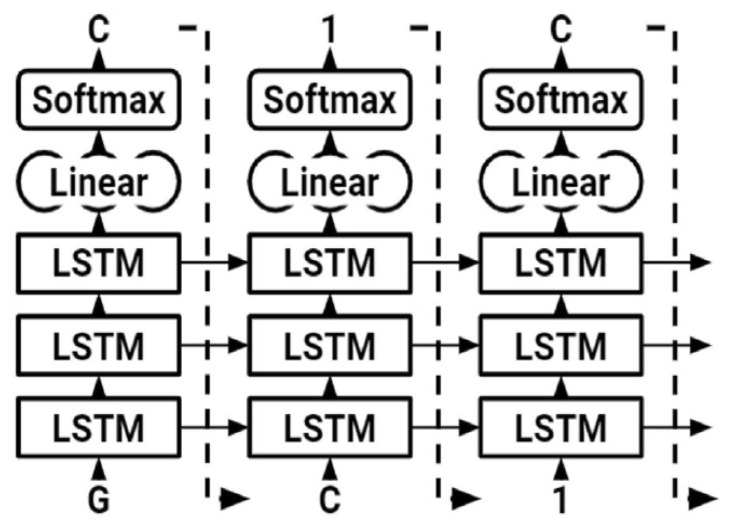
LSTM used to generate SMILE of modified gedunin.

We employed an RNN with LSTM. The LSTM network is a kind of RNN designed to simulate long-term relationships appropriately. LSTMs are made up of cells, each of which has three neural network layers known as gates (forget, update, and output gates). These gates decide which data to keep in an extra cell-state. The cell state traverses the whole network; hence, the hidden state of an LSTM serves as short-term memory, although the cell state serves as long-term memory. LSTM was used to generate SMILE of modified gedunin (Figure 7). As shown in (Figure 8) to begin, the character “G” was entered, which started up the hidden and cell states. The network started sampling symbols one by one until the last character, “\n,” was created. To generate the output logits, a dense layer was added after the LSTM cells. After the LSTM cells, a dense layer was utilized to generate the output logits, after which they were transformed to probabilities using a Softmax pro 7.1 layer throughout sampling. The model was built with Pytorch, a major Python ML package. During training, molecules were sampled from the model to assess progress (Figure 8); the model learned fast to create acceptable molecules. The amount of data utilized to train neural models determined their performance. It is feasible to augment data collection in some ML fields by altering training samples.

To validate that the model was running in the exact chemical space as the initial training data, we examined the molecules created by the LSTM with those found in the initial training data. The features of the molecules in the given dataset and those created by the LSTM overlapped considerably, demonstrating that the model could correctly replicate but not exactly copy the training data. To illustrate the ten properties (interleukin-23 receptor inhibitor, adenosine kinase, and mitotic spindle assembly protein MAD2B inhibitor, antiproliferative activity, cytotoxicity against human HCC366 cells, anticancer activity against human T47D cells, growth inhibition of human MCF7 cells, antiprogestational activity, anti-inflammatory activity, and anti-tumor activity), we used principal component analysis (PCA).

LSTM does not have critical connections such as conventional feed for neural organizations. It can not only cycle single-focused information (such as photos), but also entire information groups (such as discourse or video). For example, LSTM is suitable for messages such as unsegmented penalty recognitions, speech identification, and detection of an anomaly in network traffic or IDSs (interruption location systems).

### 3.9. Convolutional Neural Network (CNN)

**Figure 9:**
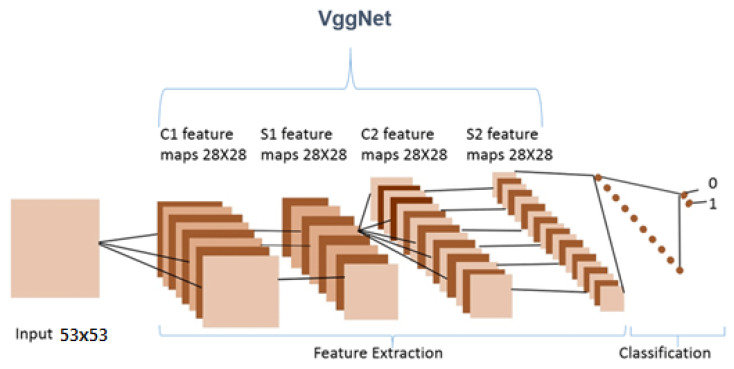
The architecture of CNN layers for feature extraction and classification [35]. The classifier is typically made up of fully connected layers.

CNNs are fundamental neural organizations, which are extremely layered, and the large majority of them feature comparable essential capacities, including convolution layers, pooling, and grouping layers (Figure 9). CNNs basically contrast each other by introducing and grouping these key layers and also by developing an organizing plan. In the first place, the information images are specifically pre-processed as standard. The input stream is then transferred into many convolutionary layers with pooling layers in which extraction and recurrence are highlighted. The basic features build continuously in a constructive way. Each highlight is afterward partially consolidated and the following highlights each signify a design aspect of the class called long term; these highlights are transferred to the totally related layer and this layer gives a measurement of the order.

#### 3.9.1. Pre-Processing Layer

Several information images must be loaded from the information layer. A few pre-processing activities, including measurement and standardization, are necessary before this. However, CNNs ask for far fewer pre-processing tasks than other neural networks. Another important factor in removing uninteresting contrasts is the basic pre-processing layer.

#### 3.9.2. Convolutional Layer

By eliminating interesting highlights from it, a convolutionary layer consolidates the contribution, including maps due to different component locators, as illustrated before. In the first convolutionary layer, the neurons scrub straight as edges. In the accompanying convolutionary layers, neurons can collect data to obtain a larger image of the image, therefore making a high-demand distinction between them [36,37]. Every turning bite is equipped for highlighting the removal across the information plane, although neurons are allocated to diverse parts of the information photographic plane to provide the part maps in the same size of the responsive field.

#### 3.9.3. Convolution

Each convolutionary layer has several limits such as the information size, bit size, guide stack depth, zero coiling, and step.

#### 3.9.4. Activation

In all cases, an actuation should occur after the weighted whole and a propensity. Apart from pure perceptrons, the simple direct mix of information is predicted to break apart and make it viable for a neuronal organization to become the general approximation of non-stop capabilities in a Euclidean environment. However, Jarrett et al. eventually brought the Corrected Linear Units (ReLUs) to CNNs to enhance the display. For some time now, Xavier Glorot et al. have pointed out that the very strong non-linearity, differentiability, and insufficient component of ReLU must be acknowledged. Ultimately, ReLUs are generally recognized for initiating convolutional yields.

#### 3.9.5. Pooling Layer

Pooling or sub-sampling includes several kinds of activities such as wide pooling, pooling, and so on; pooling usually interferes with a few convolutionary levels [38]. The most-often used pooling techniques in CNNs are max-pooling and normal pooling. Pooling offers CNNs several benefits. In particular, grouping goals to prevent overcrowding by concentrating information nearby with a window that decreases the dimensionality of the information. Dimensional information decreases further assistance to reduce estimates. In addition, pooling achieves invariance including interpretation, pivot, and scale since a few distinctions or scaling do not distinct after appropriate pooling.

#### 3.9.6. Classification Layer

The group layer is the top tier of the organization, where the final enmeshed element is collected and a segment vector is returned, where each column is directed to a class. Most clearly, each component of the yield vector deals with the probability assessment for each class and the number of components [39]. While convolutions and information about the crude map in the element space are linked, the arrangement layer results in an example space presentation, which offers a remarkable exhibition of order. Unique CNNs generally end up with a fully related layer. The entirely linked characterization layer is a heritage, which goes out to AI with “maintain extraction and grouping”. All high requirements are joined and weighed by complete connections of neuronal contributions to achieve the spatial change.

### 3.10. Transfer Learning

**Figure 10:**
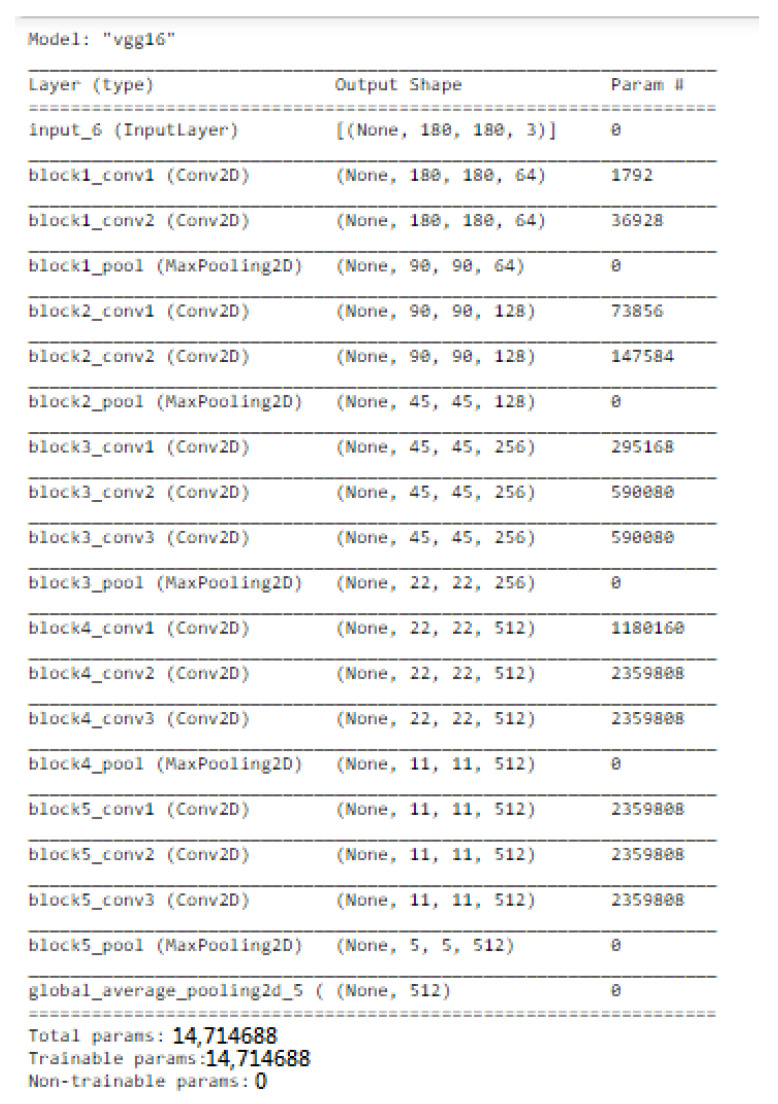
Transfer Learning vgg16 was applied for molecular feature extraction.

**Figure 11:**
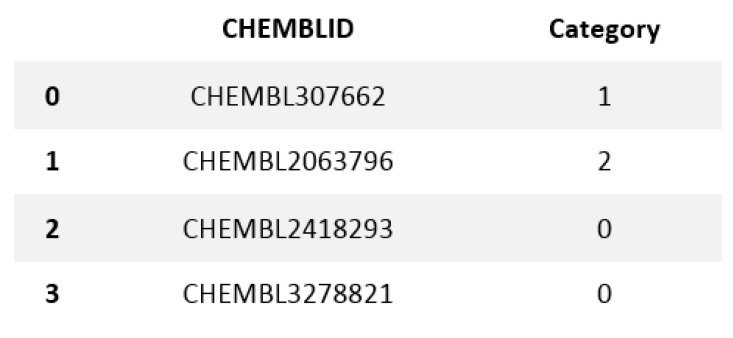
New molecule created with clusters assigned.

A deep CNN model was developed using transfer learning to increase CNN’s expectation accuracy (Figure 10). The chembl dataset was used for the pre-workout of the model. The model was pretrained on 5021 compounds and the particular atomic characteristics were removed. After the preparation of the scenes, a flattened layer trapped through a dense layer, a drop-out layer, and a single node dense cap were used to shorten and subtract the pre-trained models. LSTM was used to convert the modified gedunin SMILE into an image, which was further classified in cluster 2 (Figure 11)

### 3.11. K-Means Clustering

In this paper, K-means was applied to identify molecular clusters. Let us suppose that we needed to visualize the M layers of VGG16. The output of this layer could have the shape (A, B, C). As a result, we obtained Z∗C dimensional vectors (for one specific image), where Z=A∗B. We can already implement K-Means to these vectors (four clusters) and color this image based on the clustering result. Because the A∗B feature map indicates a scaled-down version of this image, we simply colored the corresponding image region for each of these vectors.

The K-Means partition algorithm is a bunch-like method and J.B. MacQueen proposed it. This solo algorithm is typically used in the mining of information and for example recognition. This algorithm is defined as targeting group execution files, square error, and error. This method tries to find K divisions to show a certain standard to seek the optimization outcome. Right off the bat, a few spots were selected to address the central points of the underlying group (usually, the primary K pay points are selected in a case for the underlying bunch point of convergence); furthermore, the remaining example dabs were assembled in the middle of the central focus, based on the lower distance, at that point, we obtained the underlying arrangement. K-Means isolation-dependent algorithm is a kind of bunch algorithm and benefits from speed, skill, and speed. This technique, however, depends greatly on introductive specks and the difference to start selecting instances, which always yields different results. In addition, this track-dependent algorithm uses the slope method constantly to get extremely high. The search is constantly performed in the angle technique along the direction where energy is diminished, which results in the lesser point of the neighborhood when the underlying convergence point is not legitimate. A total of 4 clusters were chosen to divide molecules into clusters based on comparable functions or characteristics based on a silhouette score (Figure 1). New molecules were created in the shape of SMILES and allocated clusters in Figure 11.

## 4. Conclusions

In this work, we applied an LSTM with RNNs to predict the properties of molecules of modified gedunin. According to the outcomes and analysis, it is clear that DL (CNN, transfer learning) and K-means algorithm can be applied to predict properties of molecules of newly generated compounds using a SMILE as raw data. More than 5000 molecules for training were utilized and vggnet for extraction of features was employed in this paper. Based on a resemblance of characteristics based on LSTM/RNN, clusters were developed with pre-exercise data and the newly generated SMILE of modified gedunin. During training, molecules were sampled from the model to assess progress; the model learns fast to create acceptable molecules. The quantity of data utilized to train neural models determine their performance. To validate that the model was running in the exact chemical space as the initial training data, we examined the molecules created by the LSTM with those found in the initial training data. The features of the molecules in the given dataset and those created by the LSTM overlap considerably, demonstrating that the model can correctly replicate but not exactly copy the training data.

Our current study extends the use of ML to predict the molecular properties of modified gedunin. To support this postulation, neural network clustering based on the AI picture was used and evaluated in this study. The novel chemical developed via profound learning has long been predicted on characteristics. As a result, LSTM with RNNs allows us to predict the properties of molecules of modified gedunin. In addition, every iteration of transfer learning results in a PCA projection of the molecular descriptors of created molecules. To optimize the required features, the model concentrates on and begins to uncover new areas of the chemical space. The chembl dataset was used for the pre-workout of the model. The model was pre-trained on 5021 compounds and the particular atomic characteristics were removed. After the preparation of the scenes, a flattened layer trapped through a dense layer, a drop-out layer, and a single node dense cap were used to shorten and subtract the pre-trained models. The total accuracy of the suggested model is 98.68%. It is obvious that the accuracy of the molecular property prediction of modified gedunin research is promising enough to evaluate extrapolation and generalization.

The model suggested in this research requires just seconds or minutes to calculate, making it faster as well as more effective than existing techniques. In short, ML can be a useful tool for predicting the properties of modified gedunin molecules.

The Cheminformatics app used to conduct these analyses is in ongoing development and is still in pre-release form. In the future, new models and functionality will be introduced.

## Data Availability

The datasets generated during and/or analyzed during the current study are available from the corresponding author on reasonable request.

## Sample Availability

Samples of the compounds are not available from the authors.
